# Neighborhood Deprivation Negatively Impacts Children’s Prosocial Behavior

**DOI:** 10.3389/fpsyg.2016.01760

**Published:** 2016-11-14

**Authors:** Lou Safra, Teodora Tecu, Stéphane Lambert, Mark Sheskin, Nicolas Baumard, Coralie Chevallier

**Affiliations:** ^1^Laboratoire de Neurosciences Cognitives, INSERM U960, Département d’Études Cognitives, Ecole Normale Supérieure – PSL Research UniversityParis, France; ^2^Faculty of Philosophy, University of BucharestBucharest, Romania; ^3^Institut Jean-Nicod, CNRS UMR8129, Département d’Études Cognitives, Ecole Normale Supérieure - PSL Research UniversityParis, France

**Keywords:** prosociality, poverty, deprivation, behavioral ecology, SES, dictator game

## Abstract

Children show stronger cooperative behavior in experimental settings as they get older, but little is known about how the environment of a child shapes this development. In adults, prosocial behavior toward strangers is markedly decreased in low socio-economic status (SES) neighborhoods, suggesting that environmental harshness has a negative impact on some prosocial behaviors. Similar results have been obtained with 9-year-olds recruited from low vs. high SES schools. In the current study, we investigate whether these findings generalize to a younger age group and a developing country. Specifically, we worked with a sample of thirty-nine 6- to 7-year-olds in two neighborhoods in a single city in Romania. Using a “Quality Dictator Game” that offers greater resolution than previous measures, we find that children living in the harsher neighborhood behave less prosocially toward a stranger than children living in the less harsh neighborhood.

## Introduction

Prosocial behaviors and motivations emerge early in development, with children in their second year already motivated to provide information to others, to spontaneously pick up objects to help others, and to comfort others in distress (e.g., Eisenberg and Miller, 1987; Warneken and Tomasello, 2006, 2009; Dunfield et al., 2011). Although more costly forms of prosociality, like spontaneous sharing, typically emerge later (Smith et al., 2013; Sheskin et al., 2014), costly prosociality can be seen during the preschool years in cooperative contexts (Hamann et al., 2011).

**FIGURE 1:**
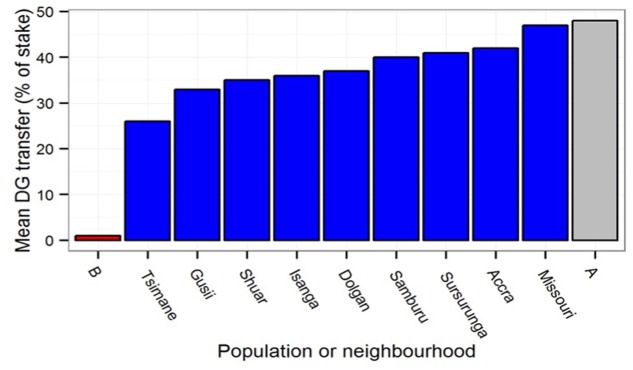
**Mean dictator game offer in the deprived (B)** and less deprived **(A)** neighborhoods in Nettle et al. (2011) compared to mean population offers from cross-cultural data in Henrich et al. (2010).

Less is known about how this development is shaped by a child’s environment. In adults, several studies have demonstrated that a behavioral ecology approach can predict part of the variability observed in the prosocial behavior of different individuals. In a study comparing prosocial behaviors in a very deprived and a more affluent neighborhood of Newcastle-upon-Tyne (UK), Nettle et al. (2011) found that participants from the very deprived neighborhood gave substantially less in a dictator game than participants from the more affluent neighborhood. Strikingly, the difference in prosocial behavior in the dictator game in different neighborhoods of this *single* city was an order of magnitude larger than the largest differences found in previous research on differences across cultures (Henrich et al., 2010). This suggests that environmental harshness *within* a single culture—and not only differences *across* cultures—calibrates prosocial motivations; indeed, environmental harshness within a culture may be far more important than any cross-cultural differences (see **Figure 1**).

Consistent with these results, in another study of 20 neighborhoods in London (UK), a strong negative effect of neighborhood income deprivation on altruistic behavior was found, with letters dropped in the poorest neighborhoods having 91% lower odds of being returned than letters dropped in the wealthiest neighborhoods (Holland et al., 2012; see also Silva and Mace, 2014, 2015).

Importantly, research with adults does *not* show decreased prosocial behavior in all situations. In the studies reported by Nettle et al. (2011) for instance, there was no difference across neighborhoods in the likelihood to help in face-to-face interactions (when the experimenter dropped an object, asked for directions, or needed to make change). A potential explanation for this phenomenon is that people in harsher environments are less likely to incur a cost to assist unseen individuals (especially when those individuals are anonymous), but are just as likely as people in more secure environments to assist individuals who are nearby. This pattern of results may be explained by an analysis of the costs and benefits of acting prosocially toward others: many accounts of moral behavior indeed emphasize that prosocial behaviors that improve one’s moral reputation can give access to the long-term benefits associated with future cooperation (Barclay, 2011; Baumard et al., 2013). In harsher environments with fewer resources and less resource security, it can be dangerous to invest large amounts in one’s cooperative reputation with strangers with whom one might never interact again (Baumard and Chevallier, 2015).

In the developmental literature, several studies have found that the trajectory of prosocial behavior shows both consistency and variation across diverse cultural contexts. In a study of 3- to 14-year-olds across six diverse societies, House et al. (2013) found that *costless* prosocial behavior increased with age in each society, whereas the development of *costly* prosocial behavior showed differences starting around middle childhood (when children’s behavior started to tend toward the adult behavior prevalent in their culture). In a study of 4- to 15-year-olds across seven diverse societies, Blake et al. (2015) found that an aversion to receiving *less* than another child unfairly emerged by middle childhood in each culture, but that aversion to receiving *more* than another child unfairly emerged in a minority of the cultures, and only later in development. Such results emphasize that the emergence of certain features of prosocial behavior is influenced by the environment.

The most directly relevant developmental study is one in which (similar to the study of adults within one city by Nettle et al. (2011), children within a single culture showed differences depending on local environmental harshness. Benenson et al. (2007) had 4-, 6-, and 9-year-old English children from low and high socio-economic status (SES) schools play a dictator game (with 10 stickers and an unknown recipient). They found that 9-year-olds (but not 4- and 6-year-olds) from high SES schools behaved more prosocially than their lower SES counterparts.

In the current study, we set out to replicate and extend the results by Benenson et al. (2007) and Nettle et al. (2011). As in previous research, we compared prosocial behavior from two samples within a single city, and the samples were recruited from neighborhoods that contrasted in deprivation level. Importantly, the participating schools were matched in terms of facilities, distance to the city center, and number of teachers. In contrast with both previous studies in which participants lived in a developed country (England), our research investigated whether similar results would be obtained in a developing country (Romania). A second extension of our study is that we gathered socio-demographic data on each child and their family to ensure that we would have a fine-grained measure of children’s social environment. Finally, we used a different method, the “Quality Dictator Game” (adapted from Sheskin et al., 2016), that might be more adapted to young children, potentially allowing us to detect differences at younger ages than previous research.

The Quality Dictator Game investigates how children allocate windfall resources that vary in quality. The Quality Dictator Game allows for an additional analysis that is not possible in studies using a pool of identical resources (e.g., 10 stickers in Benenson et al., 2007). Whereas studies that use identical resources can only measure variation in the *number* of resources allocated by different participants, the Quality Dictator Game can measure variations in the value of allocated resources. Specifically, children first allocate four toys of varying quality between oneself and another child, then complete a distractor task, then rank 12 toys (including four toys identical to the ones used in the allocation task). This design produces a “transfer score” for each child based on the value of the toys she kept (subtracted from score) compared to the value of the toys she transferred to the other child (added to score). Taking children’s individual preferences into account to calculate this transfer score thus provides a fine-grained measure of children’s prosocial behavior.

Previous research on the impact of SES and neighborhood deprivation on prosocial behavior has generally used income as a criterion to define both low/high SES and deprived/affluent neighborhood. In our study, we made sure that both SES and neighborhood status were consistent: we thus included children in the deprived group only if their parents reported earning less than the Romanian minimum monthly wage and if they lived in a deprived neighborhood. The reverse criteria were used in the non-deprived group. We predicted that children from deprived environments would have lower transfer scores than children from non-deprived environments. We had no specific predictions regarding gender, IQ, or ethnicity, and included them to control for potential effects of these variables on transfer scores.

## Materials and Methods

### Participants

Children were recruited from two schools, both situated within the same city of Slatina (Romania). The schools are located 2.2 km away from one-another and are both about 1 km away from the city center. They are comparable in terms of number of students, qualified teachers, and facilities. They differ in SES: one school (School A) is located in a mostly middle-SES neighborhood and the other (School B) is situated in a very deprived neighborhood (**Table 1**). We excluded from the analysis children from School A whose parents’ monthly income was lower than 850 lei and children from School B whose parents’ combined income was higher than 850 lei. The threshold was fixed *a priori* to 850 lei as it was the minimum monthly wage for an employee in Romania at the time we conducted the study, See Monitorul Oficial, Partea I nr. 776 din 12.12.2013. Parents were asked what their combined income including social aids was, with only two response options: “less than 850 lei,” “more than 850 lei.” This value is approximately $200.

**Table 1:** Differences between School A and School B.

	School A	School B
School values	• Integrative education	• Competence
	• Peace	• Initiative
	• Team work	• Competition
	• Cooperation	• Innovative spirit
	• Self-development and affirmation	• Sincerity
		• Tolerance
Number of classrooms and teachers	• 18 class-rooms	• 22 class-rooms
	• 22 teachers	• 25 teachers
Material resources	• TVs, copy machines, printers, video, projector, scanners, digital cameras	• TVs, copy machines, printers, video, projector, scanners, digital cameras
	• Library (11,500 volumes)	• Library (7,804 volumes)


*The data was collected from each school’s Secretary Department and it is valid for the 2013–2014 school year.*

All the results found by contrasting the two schools were confirmed using parental income (above or below the minimum wage) as the grouping variable (see Appendix 1 in Supplementary Material).

We tested 41 children aged 6–7 years (*M* = 6.9 years, *SD* = 0.43 years, range = 6.1–7.9): 18 children were girls (44%), and 20 identified with the Roma minority (49%). A minimum target of 20 participants per group was pre-planned based on the number of 6- to 8-year-olds in the low-SES school; the exact number was determined by scheduling constraints and by the number of parental consent forms we received. One child from School A and one from School B were excluded, as they did not meet our pre-determined parental income criterion. Our remaining sample thus contained 19 children from School A (*M* = 6.9 years, *SD* = 0.40, range = 6.1–7.4), with seven girls (37%) and 16 children declaring being Roma (84%), and 19 children from School B (*M* = 6.9 years, *SD* = 0.40, range = 6.1–7.9) with 10 girls (53%) and four children declaring being Roma (21%). There was no significant difference between schools in term of gender (Fisher’s *t*-test: *p* = 0.32), but children from School B were significantly more likely to declare belonging to the Roma minority (Fisher’s *t*-test: *p* < 0.001).

### Ethics

The study was approved by the schools’ management team and by the School Inspectorate. Parents signed a written informed consent form for them and their children to participate in the study and for their anonymized data to be included in the analysis. Children provided verbal assent at the start of the procedure. This research is the result of a collaboration between a French and a Romanian university, both of which require no formal approval from an ethics committee for non-invasive research. All study procedures were consistent with the Declaration of Helsinki.

### Procedure

Each child was tested individually in a quiet room, close to their own classroom. The child and the experimenter sat across from each other at an empty table. The experimenter introduced the activity by saying: “Today we will play a few games and I will ask you to help me do some tasks.” The experiment consisted of three parts: a toy distribution task, the administration of the Raven’s Color Progressive Matrices test, and the toy ranking task. Additionally, parents completed a questionnaire regarding the participating child’s family and life conditions.

#### Toy Distribution Task

The toy distribution phase began by showing the four toys to be distributed: a yellow whistle, a white ping-pong ball, a pencil, and an arrow sticker. A pretest with a different group of children and a larger set of toys (see Appendix 2 in Supplementary Material) showed that the whistle and ping-pong ball were generally ranked among the highest value toys and that the pencil and arrow card were generally ranked among the lowest value toys. The experimenter explained that any toys put on a blue mat in front of the child would go to the child herself, whereas any toys put on a yellow mat opposite the child would go to another child “who I will see next week, who you don’t know, and who you are not going to meet.” We then asked the child: “So, where do you want to put the toys?” After the child allocated the four toys, they were put in two envelopes, one for the child and one for the “other child,” and the envelopes and the placemats were put to the side for the remainder of the study.

#### Raven’s Color Progressive Matrices Test

The Color Progressive Matrices test (Raven et al., 2003) is a non-verbal test assessing reasoning skills and providing an accurate estimate of IQ (Mackintosh, 1998). It comprises 36 items presented in increasing order of difficulty within each of three 12-item sets. The Raven’s Color Progressive Matrices test provides a single raw score that is then converted to a percentile based on normative data. This test was included in our procedure so that we could control for individual differences in reasoning skills, and to provide a distractor task in between allocating toys in the toy distribution task, and judging the value of those toys in the toy ranking task. This long and challenging distractor task was chosen to limit the possibility that children would recognize that 4 out of 12 toys in the ranking task that were identical to the one they distributed in the toy distribution task, and have their ratings modified by endowment effects (Kahneman et al., 1991). The test took 15–30 min to administer.

#### Toy Ranking Task

The toy ranking task began by asking for the child’s assistance in sorting 12 toys between “cool” and “not so cool” piles, on a green and an orange rectangle placing mats that were placed side-by-side. The set included four toys that were identical copies of the ones that the child had previously allocated in the toy distribution part of the study, as well as eight other toys (a balloon, a wooden building brick, a green plastic frog, a white rubber, a red paper flower, a green rubber band, a yellow car card, and a colorful spring). After sorting the toys into the two groups, the child was asked to rank-order them in each group. This was accomplished first for the “cool” pile, and then for the “not so cool” pile, by choosing first the “best” toy in the group, then the “next best” toy, then the “next best” toy, and so on. At the end of the toy ranking task, children were thanked for their participation and given the toys they had chosen to keep for themselves in the toy distribution task.

#### Questionnaire for Parents

One of the child’s parents completed a questionnaire (see Appendix 3 in Supplementary Material) consisting of 19 items distributed between two dimensions: general information about the family structure, age, ethnicity, and educational level of the parents, and general information regarding the child’s life conditions (i.e., nutrition, number of rooms of the house, home utilities, means of transport to reach school, family monthly household income). The estimated time to complete the questionnaire was 5–7 min. Questionnaires were completed with the help of the experimenter when the parent was illiterate.

### Calculating the Transfer Score

**FIGURE 2:**
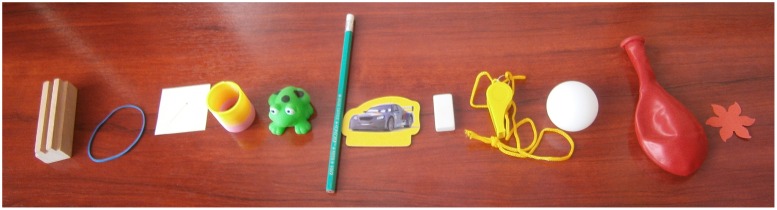
**Sample of toy ranking.** The star (right) is ranked highest and the wooden block (left) is ranked lowest.

The data about each child’s preferences collected in the toy ranking task allowed us to calculate a “transfer score” that measures the relative value of the toys allocated to each person for each child. The transfer score for each child was calculated in two steps. First, we assigned each of the 12 toys a value based on the child’s rank-ordering of the toys. The best toy got 12, the second 11, etc. Thus, the value is 13 minus the rank. We then calculated the child’s transfer score by adding the values of any toys transferred to the other child and subtracting the values of any toys kept for self. For example, here is the calculation for a child who had ranked the ping-pong ball third and allocated it to self, ranked the whistle fourth and allocated it to self, ranked the pencil seventh and allocated it to other, and ranked the sticker tenth and allocated it to other (see **Figure 2**): score = - (13 - 3) - (13 - 4) + (13 - 7) + (13 - 10) = -10 - 9 + 6 + 3 = -10. A negative transfer score indicates taking an advantage for oneself, a positive score indicates giving an advantage to the other child, and a score of 0 means a perfectly equal distribution.

## Results

### Comparisons between Schools

We first compared the environments experienced by children from School A and School B based on the data provided by the children’s parents in the Parental Questionnaire (Supplementary Material).

#### Distance from School

Children from School A and School B lived at a similar small distance (in minutes) from their school [School A: *M* = 11.47, *SD* = 6.41; School B: *M* = 10.95, *SD* = 5.98, independent *t*-test: *t*(36) = 0.26, *p* = 0.80].

#### Family Structure

Children from School B had younger parents [School A: *M* = 34.8, *SD* = 4.5; School B: *M* = 30, *SD* = 4.12, independent *t*-test: *t*(36) = 3.38, *p* < 0.002] and more siblings [School A: *M* = 0.47, *SD* = 0.61; School B: *M* = 1.8, *SD* = 1, independent *t*-test: *t*(36) = 4.95, *p* < 0.001]. No parents in School A were divorced or separated while 32% of the parents in School B were. Parents in School A were significantly less likely to be separated or divorced than parents in School B (Fisher’s *t*-test: *p* = 0.020, odds ratio, OR = 4.95).

#### Nutrition and Material Living Conditions

Children in School B lived in more crowded houses, with more people per room [School A: *M* = 1.84, *SD* = 0.66; School B: *M* = 2.27, *SD* = 0.46, independent *t*-test: *t*(36) = 2.33, *p* < 0.026]. All children in School A ate meat at least once a week, while all children from School B ate meat at most once a month. All parents in School A declared having both electricity and a washing machine at home, while 79% of the parents in School B declared having no electricity and no washing machine at home (Fisher’s *t*-test: *p* < 0.001, OR = 21.59). All parents in School A owned a refrigerator, while 32% of children in School B had no refrigerator at home (Fisher’s *t*-test: *p* = 0.020, OR = 4.95).

#### Employment

Mothers in School A were significantly more likely to have a job at the time of the experiment (89%) than mothers in School B, who all declared being a stay-at-home mother (Fisher’s *t*-test: *p* < 0.001, OR = 27.25). All fathers in School A were employed, ran a business or worked as a free-lancer, while 84% of the fathers in School B declared being unemployed. Fathers in School A were significantly more likely to work than fathers in School B (Fisher’s *t*-test: *p* < 0.001, OR = 24.29).

#### Parental Education Level

Mothers in School A were significantly more likely to have at least a high-school level of education (100%) than mothers in School B (37%) (Fisher’s *t*-test: *p* < 0.001, OR = 14.74). Fathers in School A were also more likely to have at least a high-school level of education (100%) than fathers in School B (32%) (Fisher’s *t*-test: *p* < 0.001, OR = 27.25).

Overall, this descriptive analysis confirms that children from School A and School B live in drastically different environments in terms of deprivation: children in School B have younger parents, with a lower education level, a lower combined income, and a higher chance of being unemployed. Children in School B also have more siblings, they live in more crowded houses, and they experience important material poverty and poorer access to food items like meat. Note that all the families in our sample ate meat at least occasionally, so that rare frequency of access to meat was not due to some families choosing to be vegetarian but rather due to meat being an expensive food item.

This descriptive analysis thus confirms that School A is situated in a middle SES neighborhood and School B is situated in a low SES neighborhood.

### Toy Distribution Task

#### Transfer Score

**FIGURE 3:**
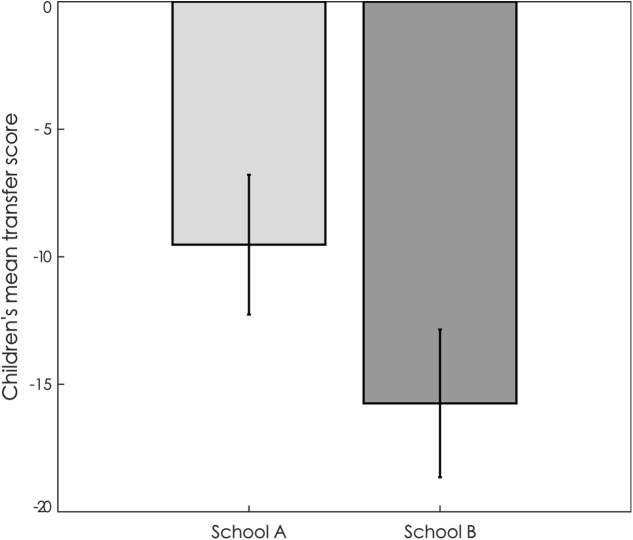
**Mean transfer score in children from School A and School B.** Error bars represent standard errors to the mean.

We followed the same analysis plan as Sheskin et al. (2016), on which our experiment is based, and compared the transfer scores of the children from two schools. Because two outliers were identified in our dataset, we used robust linear regression to analyze the transfer scores (outliers: *N* = 1 in School A with transfer score of +20; *N* = 1 in School B with a transfer score of +23). The mean transfer score was -9.53 in children from School A (*N* = 19, *SD* = 11.95, range = -29 to +20) and -15.75 in children from School B (*N* = 20, *SD* = 12.95, range = -30 to +23). This analysis revealed that children from the school situated in the very deprived neighborhood transferred significantly less to a stranger than children from the school situated in the less deprived neighborhood [*t*(37) = -2.72, *p* = 0.010; **Figure 3**].

We then ran a robust linear regression on children’s transfer score, taking school, IQ, ethnicity, and gender as factors. In this analysis, the effect of school was still present as a trend [*t*(34) = -1.84, *p* = 0.075], while gender [*t*(34) = 1.27, *p* = 0.210], IQ [*t*(34) = -0.99, *p* > 0.250], and self-declared ethnicity [*t*(34) = 1.06, *p* > 0.250] were not significant predictors of children’s transfer scores in this regression. Importantly, even if the two schools significantly differed in their ethnic compositions [χ^2^ (1, *N* = 39) = 11.29, *p* < 0.001] and in their mean IQ [*t*(37) = 2.16, *p* = 0.038], no multicollinearity problem was evidenced in the regression taking school, gender, self-declared ethnicity, and IQ as factors (maximal variance inflation factor = 1.77, see Hair et al., 2006). In summary, even after controlling for IQ, gender and self-declared ethnicity, the most deprived neighborhood was associated with lower transfer score than the less deprived neighborhood.

#### Average Transfer Score

The two schools differed in their ranking of one of the four toys used in the task (the whistle). Average ranking of the whistle was lower in School A than in School B [*t*(37) = -2.24, *p* = 0.031; no significant difference for the other three toys: all *t*(37) < 0.72, all *p* > 0.250]. It is possible that this difference affects the comparison of the transfer score between the two schools. To rule out this possibility, we used the average value of each toy to compute an *average transfer score* for each child. This analysis is less reflective of individual differences in toy valuation, but ensures that significant differences between School A and School B are due to differences in toy distribution rather than toy ranking (since the same average ranking is used across all children in the study).

The conclusions from this additional analysis are identical to the conclusions from our pre-planned analysis. Using the average values of the toys (arrow card: *M* = 1.97, *SD* = 1.40; ping-pong ball: *M* = 7.26, *SD* = 2.27; pencil: *M* = 5.21, *SD* = 2.18; whistle: *M* = 7.67, *SD* = 2.35), we found that the mean average transfer score was -10.12 in children from School A (*N* = 19, *SD* = 11.84, range = -22.10 to +22.10) and -15.84 in children from School B (*N* = 20, *SD* = 12.23, range = -22.10 to +22.10). A robust linear regression revealed that the Average transfer scores of children from School B were significantly lower than those from School A [*t*(37) = -5.33, *p* < 0.001]. Importantly, as for the individual transfer score, this effect was still present as a trend after controlling for children’s IQ, self-reported ethnicity and gender [*t*(34) = -1.71, *p* = 0.096]. Children from the school situated in the very deprived neighborhood thus transferred less to a stranger than children from the school situated in the less deprived neighborhood, whether or not we used individual value scores or the same value for each child.

#### Number of Toys Given

It is also possible to supplement our pre-planned analysis of transfer scores (the defining feature of the Quality Dictator Game), with a more standard analysis of how many toys were kept and how many were transferred. Thus, we compared the average number of toys given away by children from School A (*M* = 1.37, *SD* = 1.16, range = 0–4) and from School B (*M* = 0.65, *SD* = 1.09, range = 0–4). A robust linear regression on the number of toys given revealed that children from School A gave more toys on average than children from School B [*t*(37) = -2.12, *p* = 0.041]. Therefore, this more standard analysis confirmed the results obtained in the previous analyses.

## Discussion

Consistent with what Nettle et al. (2011) found with English adults and what Benenson et al. (2007) found with English 9-year-olds, we showed that variation in deprivation within a single city in a developing country influences the prosocial behavior of 6- to 7-year-olds toward an anonymous stranger. The relationship between neighborhood deprivation and children’s prosociality toward a stranger held when controlling for children’s IQ, self-declared ethnicity, and gender.

Furthermore, two *post hoc* analyses converge with the pre-planned analyses. First, calculating children’s transfer scores based on the average ranking of the toys did not change the results, suggesting our results were not driven by children valuing the toys they distributed in the toy distribution task differently when ranking them later among others (e.g., because of an endowment effect). Second, a comparison of the number of toys given showed a trend toward children from School A giving more toys than children from School B, showing that children from the middle SES neighborhood gave away more toys than children from the more deprived neighborhood.

Our study contributes to a better understanding of the impact of a harsh social environment on the development of prosocial behaviors toward strangers. However, it is important to note that the effects of environmental harshness on prosociality may not be linear. Both our results and the results of Benenson et al. (2007) are based on children living in low- and middle-SES environments; different results might be found when comparing middle- and high-SES environments. In fact, recent results by Miller et al. (2015) suggest that upper-SES children may behave *less* prosocially than middle-SES children. In their study, 4-year-old children from middle-SES backgrounds shared more of 10 tokens with a sick child than did high-SES children. One candidate underlying mechanism could be that competitiveness is higher in upper- vs. middle-SES children (e.g., Knight and Kagan, 1977; for a similar argument in teenagers, Buunk et al., 2013). Another possibility is that the impact of the child’s social environment on prosocial motivation depends on the context. Children living in a harsh social environment might thus behave less prosocially in some contexts and more prosocially in others. In line with this idea, the impact of social status on prosocial behavior varies in adults. For instance, *noblesse oblige* can lead higher status people to behave more prosocially to defend their status, and higher resources associated with higher status can make prosocial behaviors less costly for the individual. Yet, being high status also gives leverage over others and decreases the costs of behaving less prosocially so that depending on the context, high status will lead to lower or higher prosociality (for a review, see Kafashan et al., 2014).

Another possible explanation of Miller et al.’s (2015) results is that the recipient of their dictator game was described as “a sick child” rather than an unknown child (as in our experiment and Benenson et al.’s 2007 study). It could be the case that SES correlates positively with prosociality toward a stranger, but that the correlation goes in the opposite direction when the recipient is not anonymous, or when empathy is involved as in Miller et al. (2015). We know of no other study where the recipient elicits empathy, but in a study by Chen et al. (2013), 4-year-old children from rural China played a dictator game with four stickers with the recipient being a friend or an unknown child. Similar to Miller et al. (2015), lower SES children gave more stickers than higher SES children in the friend condition but not in the unknown condition. In both Benenson et al.’s (2007) and Chen et al.’s (2013) studies, SES did not impact prosocial behavior by the age of 4 when the recipient was unknown, but the results of Chen et al. (2013) in the friend condition and of Miller et al. (2015) in the “sick child” condition suggest that lower SES may be associated with higher prosocial behavior in some cases. Future research could further study the development of the correlation between SES and prosocial behavior by systematically varying the identity of the recipient and by testing low, middle, and high SES children.

A number of limitations of our study should also be acknowledged: in particular, we only focused on very low and middle SES children; we had—as in previous studies—only one measure of prosocial motivation; and our sample size was too small to disentangle the effects of the different factors that together constitute a harsh social environment such as material factors (e.g., material poverty or parental income) vs. social factors (e.g., parenting style or aggressive interactions) or biological factors (e.g., toxins; see Duncan et al., 1994; Bradley and Corwyn, 2002; Evans and English, 2002; for a review: Brooks-Gunn and Duncan, 1997; Evans, 2004). It will therefore be important for future research to assess whether the effect of social background on prosociality toward strangers is robust and to test the impact of different social factors associated with harsh social environments. Further investigations would be particularly interesting to identify the pathways by which environmental harshness impacts prosociality. For instance, prosocial behavior may be directly influenced by children’s level of resources but also by the type of social interactions they are usually exposed to (e.g., competitive vs. cooperative ones) as well as by the development of their cognitive abilities.

Finally, the impact of childhood environmental harshness has been shown to extend into adulthood in other domains such as health and non-social cognition (see e.g., Case et al., 2005; Luo and Waite, 2005; Cohen et al., 2010). Future research may thus investigate whether childhood environmental harshness has a similarly long-lasting impact on prosociality, and if so, how childhood deprivation interacts with current levels of resources. This next step would be all the more informative that studies on the effect of current socio-economic status on prosociality in adults have yielded mixed results. Specifically, two articles by Piff and his colleagues have reported a positive association between low SES and prosociality (Piff et al., 2010, 2012). In these two sets of studies, American college students reporting a lower subjective SES behaved more prosocially in a dictator game (Piff et al., 2010), drivers of more expensive cars behaved less ethically than drivers of cheaper cars (Piff et al., 2012; replicated by Morling et al., 2014), and male students of higher SES reported a higher likelihood of behaving unethically in hypothetical scenarios (Piff et al., 2012; replicated by Lyons et al., 2012 and by Konigsberg et al., 2013).

In sharp contrast, a growing number of studies, including large-scale cross cultural experiments, have found that deprivation has a negative impact on prosociality (see e.g., Wilson et al., 2009; Nettle et al., 2011; Holland et al., 2012; Silva and Mace, 2014, 2015; Gomes and McCullough, 2015). In a recently published set of eight studies analyzing large and representative international samples including several thousands of participants, Korndörfer et al. (2015) found positive effects of higher SES on prosociality: based on self-reports, higher SES individuals were more likely to make charitable donations and contribute a higher percentage of their family income to charity, to volunteer, and to be helpful. Higher SES individuals were also more trusting and trustworthy in an economic game when interacting with a stranger than lower SES individuals. Getting a better understanding of how different factors associated with childhood social environment impact children and adult prosociality will advance our understanding of the causes of the great variations we observe in prosocial behaviors and motivations, and possibly help us find ways to use this knowledge to promote them.

## Author Contributions

SL, MS, TT, NB, and CC contributed to the design of the study; TT did the data collection and anonymized the data; LS, SL, TT, MS, and CC contributed to the data analysis; and LS, SL, TT, MS, NB, and CC contributed to the writing of the paper.

## Conflict of Interest Statement

The authors declare that the research was conducted in the absence of any commercial or financial relationships that could be construed as a potential conflict of interest.
