# Supplementary material for: Neighborhood Deprivation Negatively Impacts Children’s Prosocial Behavior
Source: Front Psychol. 2016 Nov 14;7:1760. doi: 10.3389/fpsyg.2016.01760 (PMC5107739; doi:10.3389/fpsyg.2016.01760)
Supplement: Supplementary file 1 [file Table_1.docx]

Appendix 3. Questionnaire for parents

**FOR PARENTS**

**A. GENERAL INFORMATION**

7 . NUMBER brothers / sisters of the child: _____________________________________

8 . MARITAL STATUS. Currently you are : (Please circle the letter corresponding to your marital status)

1. Married; b.Single; c. Separated; d. Divorced; e. Widow/er; f. Living with a partner

9. EDUCATIONAL LEVEL of the parents (Please circle the number corresponding to the last level of education of the mother and father of the student )

       a. MOTHER : 1 . No school 2. Primary 3. Secondary 4. High school 5. Vocational school 6. Post high school 7. Higher Education

        b FATHER : 1 . No school 2. Primary 3. Secondary 4. High school 5. Vocational school 6. Post high school 7. Higher Education

10. EMPLOYMENT. Currently you are: (Please circle the letter corresponding to your current employment status)

a. Full-time employee b. Part-time employee c. Internship d. Unemployed e. Retired f. In trials g. Housewife h. Freelancer i. I run my own bussiness

11 . If you have a husband / wife/ partner , he /she is :

a. Full-time employee b. Part-time employee c. Internship d. Unemployed e. Retired f. In trials g. Housewife h. Freelancer i. I run my own bussiness

**B. GENERAL INFORMATION ABOUT THE CHILD’S LIFE CONDITIONS**

1. Number of people living in the same house with the child: _______________________________________

2. Number of rooms (the living quarters) of the house where the child lives: _______________________

3. Do you own: (tick the appropriate response to the situation of the child)

| 5. | Refrigerator |  |
| --- | --- | --- |
| 7. | Washing machine |  |
| 8. | Electricity |  |

4 . Please estimate the average time which the child has to spend in order to cover the distance between home and school: .............. ( minutes).

7. Tick the appropriate box according to the frequency with which your child consumes the following foods:

|  |  | Daily | Two/three times per week | Weekly | Mounthly | Rearly than once a month |
| --- | --- | --- | --- | --- | --- | --- |
| 3 | Meat |  |  |  |  |  |

8 . ADDING UP YOUR FALILY’S MONTHLY HOUSEHOLD INCOMES (salary, pension, child allowance, welfare and other finacial rewords or aids), IS IT

1. Below 850 lei? € Yes € No
2. Over 850 lei? € Yes € No
