# Supplementary material for: Neighborhood Deprivation Negatively Impacts Children’s Prosocial Behavior
Source: Front Psychol. 2016 Nov 14;7:1760. doi: 10.3389/fpsyg.2016.01760 (PMC5107739; doi:10.3389/fpsyg.2016.01760)
Supplement: Supplementary file 2 [file Data_Sheet_1.docx]

Appendix 1. Analyses using Parental Income

**Methods**

Two *Parental Income Groups* (*Low* and *Medium*) were created based on the parents’ self reported monthly income (above or below the minimum monthly wage). 21 children were included in the Low Parental Income Group (7 females) and 20 in the Medium Parental Income Group (11 females).

**Results**

*Transfer score:* As in the main analysis, we used robust linear regression to analyze the impact of parental income on children’s transfer scores. Confirming the results found by contrasting the two schools, children of the *Low Parental Income Group* had lower transfer scores than children of the *Medium Parental Income Group* (*Medium Parental Income Group*: *M* = -8.85, *SD* = 12.01, *Range* = -29 - +20; *Low Parental Income Group*: *M* = -16.52, *SD* = 13.11, *Range* = -32 - +23; *t*(39) = -3.11, *p* = .003). Moreover, this difference was still significant after controlling for gender, ethnicity and IQ (*t*(36) = -2.35, *p* = .024).

*Average transfer scores:* However, as for the main analysis, the average rankings of the whistle differed between the two *Parental Income Groups* (*t*(39) = -2.51, *p* = .016; no significant difference for the other three toys: all *t*(39) < 0.60, all *p* > .250). Therefore, following the same plan of analysis as in the main results, we computed the Average transfer score for each child using toys’ average ranking to further test that the impact of Parental Income was not due to differences in toys’ ranking. Confirming our results, children of the *Low Parental Income Group* had lower Average transfer scores than children of the *Medium Parental Income Group* (*Medium Parental Income Group*: *M* = -9.03, *SD* = 12.62, *Range* = -22.29 – +22.29; *Low Parental Income Group*: *M* = -16.26, *SD* = 12.09, *Range* = -22.29 - +22.29; *t*(39) = -5.64, *p* < .001). As for the individual transfer score, this impact was still present after controlling for children’s IQ, self-reported ethnicity and gender (*t*(36) = -2.04, *p* = .049).

*Number of toys given:* Finally, similarly to the comparison of the children from the more and less deprived neighborhood, children of the *Low Parental Income Group* gave less toys than children from the *Medium Parental Income Group* (*Medium Parental Income Group*: *M* = 1.45, *SD* = 1.19, *Range* = 0 - 4; *Low Parental Income Group*: *M* = 0.62, *SD* = 1.07, *Range* = 0 – 4; *t*(39) = -2.49 *p* = .017).

**Conclusion**

All the effects of socioeconomic status shown using school as a grouping factor were confirmed using Parental Income as the grouping variable.

Appendix 2. Pre-test

The goal of the pre-test was to validate, in Romania, a choice of items for the toys distribution task that would be as similar to the original set of toys used by Sheskin et al. (2016) in the United-States.

*School*

The pretest school is located in a medium-low SES neighbourhood, that is a residential area inhabited mostly by factory workers, with middle to low income and education. It is situated further from the citycenter, in comparison with School A and School B.

*Participants*

Nine children, aged 6 to 7 years old, (*M_age_* = 6.8 years, SD = 0.5), six female and three male, formed the sample of the pretest. The pretest took place in a third school within the same city.

*Materials and procedure:*

The Procedure was identical to the procedure used during the Toy Ranking Task (see main article)

*Scoring the Toy Ranking Task:*

Twelve points were attributed to the item considered the coolest, eleven to the second coolest, down to one point for the item considered the least cool.

## *Results:*

## The items that received the highest ratings were the balloon (*M* = 9.56, *SD* = 2.18), the paper flower (*M*= 9.34, *SD* = 2.95), the spring (*M* = 9, *SD* = 2.95), the Ping-Pong ball (*M* = 8.12, *SD* = 3.01), the whistle (*M* = 7.34, *SD* = 1.80) and the frog (*M* = 7, *SD* = 3.16). The items that received the lowest ratings were the rubber band (*M* = 3.56, *SD* = 2.06), the building brick (*M* = 4.00, *SD* = 3.20), the arrow card (*M* = 4.23, *SD* =1.56), the rubber (*M* = 5, *SD* = 2.69), the pencil (*M* = 5.34, *SD* = 3.57) and the car card (*M* = 5.78, *SD* = 4.54).

This pretest confirmed that the Ping-Pong ball and the whistle were considered high value toys in Romania, and that the Ping Pong ball could replace the bouncy ball used in Sheskin et al. (an item that would have been hard to find in Romania). The pretest also confirmed that the pencil and arrow card were considered low value toys in Romania.
